# Digit forces bias sensorimotor transformations underlying control of fingertip position

**DOI:** 10.3389/fnhum.2014.00564

**Published:** 2014-08-04

**Authors:** Daisuke Shibata, Astrid M. L. Kappers, Marco Santello

**Affiliations:** ^1^Kinesiology Program, School of Nutrition and Health Promotion, Arizona State UniversityTempe, AZ, USA; ^2^Faculty of Human Movement Sciences, Move Research Institute, VU University AmsterdamAmsterdam, Netherlands; ^3^School of Biological and Health Systems Engineering, Arizona State UniversityTempe, AZ, USA

**Keywords:** hand, psychophysics, haptics, feedback, grasping

## Abstract

Humans are able to modulate digit forces as a function of position despite changes in digit placement that might occur from trial to trial or when changing grip type for object manipulation. Although this phenomenon is likely to rely on sensing the position of the digits relative to each other and the object, the underlying mechanisms remain unclear. To address this question, we asked subjects (*n* = 30) to match perceived vertical distance between the center of pressure (CoP) of the thumb and index finger pads (*d_y_*) of the right hand (“reference” hand) using the same hand (“test” hand). The digits of reference hand were passively placed collinearly (*d_y_* = 0 mm). Subjects were then asked to exert different combinations of normal and tangential digit forces (*F_n_* and *F_tan_*, respectively) using the reference hand and then match the memorized *d_y_* using the test hand. The reference hand exerted *F_tan_* of thumb and index finger in either same or opposite direction. We hypothesized that, when the tangential forces of the digits are produced in opposite directions, matching error (1) would be biased toward the directions of the tangential forces; and (2) would be greater when the remembered relative contact points are matched with negligible digit force production. For the test hand, digit forces were either negligible (0.5–1 N, 0 ± 0.25 N; Experiment 1) or the same as those exerted by the reference hand (Experiment 2).Matching error was biased towards the direction of digit tangential forces: thumb CoP was placed higher than the index finger CoP when thumb and index finger *F_tan_* were directed upward and downward, respectively, and vice versa (*p* < 0.001). However, matching error was not dependent on whether the reference and test hand exerted similar or different forces. We propose that the expected sensory consequence of motor commands for tangential forces in opposite directions overrides estimation of fingertip position through haptic sensory feedback.

## Introduction

Dexterous object manipulation requires coordination of digit forces (Johansson and Westling, 1988b; Johansson and Flanagan, 2009) and positions (Lukos et al., 2007, 2008; Fu et al., 2010, 2011; Zhang et al., 2010; Crajé et al., 2011). It has been shown that when subjects can choose digit placement on an object, they modulate digit forces to compensate for trial-to-trial variability in digit position. This behavior is thought to be instrumental for ensuring a consistent manipulation performance and might explain humans’ ability to perform the same manipulation task despite variability in where or how the object is grasped (Fu et al., 2010, 2011). Although the mechanisms underlying digit position-force coordination are not well understood, they are likely to involve integration of visual and haptic sensing of digit position, i.e., where the digits are relative to each other and the object, and motor commands responsible for distributing forces among the digits.

To understand the sensorimotor transformations responsible for the above phenomenon of digit position-force coordination, our previous study examined subjects’ ability to match the remembered relative vertical distance between the center of pressure (CoP) of thumb and index finger pads without visual feedback of the hand (Shibata et al., 2013). This study revealed that sensorimotor transformations are more accurate for *(a)* larger vertical separations between the digits’ CoP; and *(b)* when fingertips’ vertical distance is reproduced with the same hand and at the same posture as those used when sensing the fingertip distance. It was speculated that the more accurate fingertip distance matching performance found for larger fingertip distances could have resulted from a combination of factors, including afferent responses from joint receptors and higher signal-to-noise ratio of afferent signals from skin receptors in the dorsal region of the hand, which are thought to provide proprioceptive information about digit position (Edin and Abbs, 1991; Edin and Johansson, 1995; Edin, 2001, 2004; Collins et al., 2005). Moreover, it was proposed that reproduction of fingertips’ vertical distance with the same hand and at the same posture would bypass higher-order processing of fingertip distance that would otherwise be involved with transferring remembered sensory feedback to the contralateral hand or a different hand posture.

It should be noted that in our previous study (Shibata et al., 2013) we passively positioned the subjects’ fingertips to given distances and required them to exert negligible contact forces. This was done to control for the potential effect that voluntary motor commands for positioning the digits or generating forces might have had on fingertip distance matching performance. Specifically, it has been shown that when subjects are asked to match static joint angle, voluntary motor commands for force production at a given limb posture can negatively influence matching performance by biasing the error in the direction of the attempted movement (Gandevia, 1987; Gandevia et al., 2006; Smith et al., 2009). Additionally, this perceptual bias is greater when only motor commands are available following anesthesia and paralysis (Gandevia et al., 2006) than when motor commands and afferent signals are available while muscles are paralyzed (Smith et al., 2009). These findings suggest that voluntary motor commands for force production can influence the central processing of afferent signals conveying information about limb posture. This mechanism has been proposed to operate within internal forward models whose role is to predict sensory consequences of motor actions based on a copy of motor commands and an estimate of the current state of the body (Wolpert et al., 1995; Kawato, 1999). The internally-predicted sensory consequences are then compared with incoming sensory afferent signals to estimate sensory state in the immediate future.

Previous matching tasks involving force production (Collins et al., 2005; Gandevia et al., 2006; Smith et al., 2009; for review see Proske and Gandevia, 2012) did not require the perception of relative contact points or sensorimotor transformations required by the retrieval and reproduction of remembered limb postures. Specifically, these studies required subjects to indicate which direction the finger, hand, or limb was pointing to using the opposite hand while the target body parts remained at the target location. Such a matching task could be performed using proprioceptive feedback about the joint angle or posture without having to retrieve the sensory feedback of the perceived joint angle stored in memory. However, these tasks differ from grasping and manipulation tasks where the above-described digit position-force coordination might rely on sensing the fingertips’ relative position rather than digit or wrist joint angles *per se*. Furthermore, sensorimotor control of digit forces relies on prior experience with same or similar objects (Johansson and Westling, 1984, 1988a; Gordon et al., 1993; Quaney et al., 2003). This prior experience in the form of *sensorimotor memory* persists for at least 24 h (Gordon et al., 1993).

Besides the above-described effect that digit force generation might have on perception of fingertip distance, digit force production associated with grasping and manipulation is accompanied by skin deformation of the finger pads following object contact. The resultant activation of tactile afferents provide information about the magnitude and direction of force acting on the finger pads (Birznieks et al., 2001; Jenmalm et al., 2003; Barbagli et al., 2006; Panarese and Edin, 2011; for review see Johansson and Flanagan, 2009). More importantly, the contact points at which the digits apply forces on an object could be inferred through tactile feedback from the finger pad when vision of the contacts is not available. The CoP on the finger pad is likely to shift as the tangential digit force leads to skin deformation of the finger pad. Since our previous study involved a negligible tangential digit force (less than 0.2 N; Shibata et al., 2013), the contribution of lateral skin deformation induced by a shear force on the finger pad on the accuracy of matching the relative distance between contact points remains unknown.

The gaps in the above-reviewed work raise the following question: to what extent motor commands responsible for digit force production affect subjects’ ability to transform sensory feedback of relative contact points to motor commands for placing the digits to their remembered locations? To address this question, we asked subjects to perceive and reproduce fingertip distance after a short delay using the same hand. The delay was used to introduce a memory component to the matching task similar to the above-mentioned sensorimotor memory component underlying grasping tasks. Furthermore, to prevent subjects from merely matching the pressure on the finger pad and hand posture, one subject group performed the matching task without significant digit force production when matching the remembered contact points. The present study also examined subjects’ ability to reproduce the remembered digit contact points when tangential forces of the thumb and index finger were produced in the same or opposite direction. An object manipulation may require a vertical translation and/or a rotation of a grasped object. To perform a vertical translation, the digit tangential forces are produced in the same direction, whereas these forces are exerted in opposite directions to rotate an object.

We hypothesized that (1) when the tangential forces of the thumb and index finger are produced in opposite directions, the reproduction of memorized fingertip distance would be biased toward the directions of the tangential forces exerted while perceiving and memorizing the digits placement; and (2) the magnitude of the biased error would be greater when the remembered relative contact points associated with the production of relatively large digit forces are matched while exerting negligible forces. The rationale for the first hypothesis is that voluntary motor commands for force production would distort the matched joint angle and limb position in the direction of the attempted movement (Gandevia et al., 2006; Smith et al., 2009). When the direction of digit tangential forces was the same, we expected no directional bias in matching error of the relative vertical fingertip distance. The second hypothesis is based on the expectation that matching relative contact points would be facilitated by the congruent skin deformation of the finger pad used to match the remembered points with that used to perceive and remember the relative contact points. Thus, this hypothesis implies that fingertip distance matching ability would be challenged by reproducing the remembered points while experiencing different digit forces and tactile feedback associated with skin deformation on the finger pad. To test the second hypothesis, we asked subjects to match the remembered relative distance between contact points while exerting negligible or significant force.

## Materials and methods

### Subjects

Two groups of 15 healthy subjects each participated in this study. Group 1 (11 females; mean ± SD: 23.2 ± 7.0 yrs.) participated in Experiment 1, and Group 2 (5 females; mean ± SD: 22.7 ± 4.3 yrs.) participated in Experiment 2. We used the 10-item Edinburgh Handedness Inventory (Oldfield, 1971) to assess subjects’ hand dominance. All subjects were classified as right-handed based on the mean Laterality Quotient and standard deviation (Group 1: 77.8 ± 18.9; Group 2: 78.0 ± 19.2). Subjects were naïve to the purpose of the study. Subjects gave their written informed consent according to the declaration of Helsinki and the protocols were approved by the Office of Research Integrity and Assurance at Arizona State University.

### Apparatus

**Figure 1:**
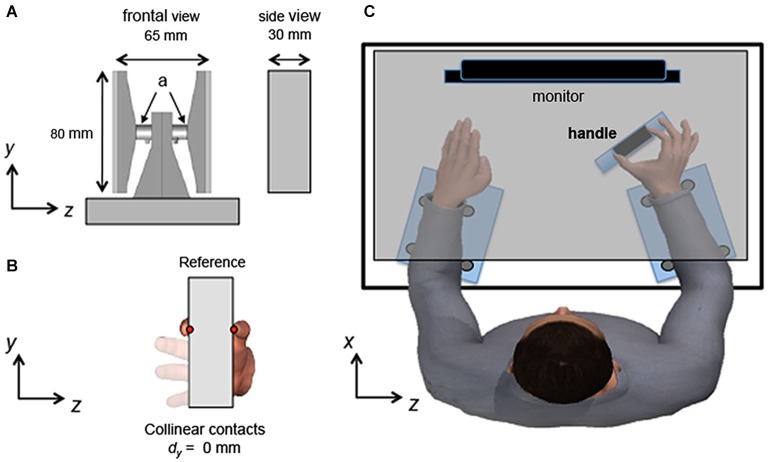
**Experiment setup. (A)** shows frontal and side views of the handle used for the study (*“a”* denotes force/torque sensors). **(B)** shows the frontal view of the handle with thumb and index fingertip center of pressure of the reference hand located at the same *y*-coordinates (vertical height relative to the base of the object) on the graspable surfaces of the handle (collinear *d_y_*). The red dots denote the **CoP** of each digit. **(C)** shows a top view of the experimental setup. The subject is shown contacting the handle with thumb and index fingertip, while the left hand was kept flat on the table. When relaxing in between trials, both hands were kept flat and relaxed. Note that the table top (gray) was opaque and prevented subjects from seeing their forearms and hands but is shown as transparent for graphical purposes only. Forearms and wrists were strapped to the table to prevent movements within and across trials while the handle was anchored to the table.

We used a custom-made grip handle to measure digit forces and CoP of the thumb and index finger pad for both Experiments 1 and 2 (Figure 1A). The sensorized handle has been described in detail elsewhere (Shibata et al., 2013). Briefly, two six-component force/torques sensors were mounted on each side of the handle (ATI Nano-25 SI-125-3, ATI Industrial Automation, Garner, NC; force range: 125, 125, and 500 N for *x-*, *y-* and *z-*axes, respectively; force resolution: 0.06 N; torque range: 3000 N•mm; torque resolution: 0.378 N•mm; Figure 1A). The vertical coordinate (*y*) of the CoP of each digit on the contact surface (red dots, Figure 1B) was computed from the force-torque sensor output. We performed calibration of each sensor by applying forces (3, 4, 5, and 6 N) perpendicular to the contact surface mounted on the sensor. This calibration revealed that the force and torque output of the two sensors could be used to compute the vertical coordinate of each digit CoP with a maximum error across all measurements and sensors of ± 1.2 mm (maximum average error ± SD: 0.3 ± 0.4 mm). Error in CoP reconstruction was similar between the two sensors and to the errors found when applying smaller normal forces (i.e., 0.6, 1.0, and 1.4 N; Shibata et al., 2013). During the experimental tasks, subjects exerted normal force with a digit within the 0.6–6.0 N range in 98% of all trials. To prevent the digits from slipping when subjects applied tangential forces up to 3.5 N, the contact surfaces of the handles were covered with 100-grit sandpaper (static friction coefficient range: 1.4–1.5).

### Experimental procedures

Subjects grasped the handle with the thumb and index finger of the right hand while sitting on an adjustable chair with both forearms resting on adjustable supports (Figure 1C). The left hand rested on the table throughout the experiment with all digits straight, adducted, and in a pronated position. Vision of forearms, hands, and the handle was prevented by an opaque tabletop on which a computer monitor was placed at subjects’ eye level (Figure 1C). The positioning of the handle and platforms was adjusted for each subject so that subjects’ digits could be placed on the handle in a comfortable posture. All subjects had similar postures of the wrist such that the wrist was semi-pronated and in a neutral posture (~0° flexion/extension and adduction/abduction). Motion of forearms and wrists was blocked by straps and rigid dowels anchored to the platform to minimize changes in posture across trials and throughout the experiment. The handle was anchored to the table to maintain a fixed position and distance relative to the hand. The experimental setup was the same across Experiments 1 and 2.

**Figure 2:**
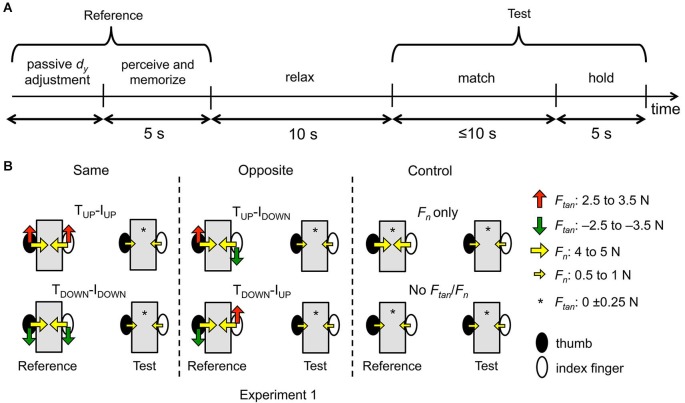
**Experimental protocol and conditions (Experiment 1). (A)** shows the time course of the experimental protocol. In the “*passive d_y_ adjustment*” phase, the subject’s thumb and index finger were passively placed by an experimenter to a collinear *d_y_* (see Figure 1B). Once the desired *d_y_* was reached and digit forces matched the desired target forces, recording of reference hand *d_y_* started for 5 s while subjects were asked to perceive and memorize the reference hand *d_y_* (“*perceive and memorize*” phase). During the “*relax*” phase, subjects were asked to relax their reference hand for 10 s, followed by the “*match*” phase in which they were asked to reproduce the remembered reference hand *d_y_* using the (same) test hand within 10 s. The test hand *d_y_* was then recorded for 5 s while subjects maintained the digit position and digit forces (“*hold*” phase). **(B)** shows the experimental conditions for Experiment 1. The thumb and index finger (filled and open ellipse, respectively) of the reference hand exerted tangential forces either in the same or opposite directions (“Same” and “Opposite”, left and middle column, respectively). In the Same condition, thumb and index finger exerted tangential forces that were both upward or downward (T_UP_-I_UP_ or T_DOWN_-I_DOWN_, respectively). In the Opposite condition, the tangential forces of the thumb and index finger were directed opposite to each other, i.e., either upward and downward (T_UP_-I_DOWN_) or downward and upward (T_DOWN_-I_UP_), respectively. In the Control condition (right column), subjects were asked to exert no tangential force while exerting large or negligible normal forces (“*F_n_* only” or “No *F_tan_*/*F_n_*”, respectively). The magnitude of tangential and normal forces was the same across these conditions (*F_tan_*: 2.5–3.5 N, *F_n_*: 4–5 N) with the exception of the “No *F_tan_*/*F_n_*” condition (*F_tan_*: 0 ± 0.25 N, *F_n_*: 0.5–1 N). The test hand in Experiment 1 exerted only negligible tangential and normal forces (*F_tan_*: 0 ± 0.25 N, *F_n_*: 0.5–1 N).

For both experiments, after subjects’ digits were passively moved *(“passive d_y_ adjustment”* phase, Figure 2A), we asked subjects to perceive and memorize the vertical distance (*d_y_*) between thumb and index CoP of the right hand (“reference” hand) *(“perceive and memorize”* phase, Figure 2A), relax for 10 s, and match it using the same hand (“test” hand) (*“match”* phase, Figure 2A). We used a 10-s delay between the *“perceive and memorize”* and *“match”* phase in our previous work to ensure memory formation (Shibata et al., 2013). We used the same delay in the present study to allow comparison with our previous work. An important difference between the present study and our previous work (Shibata et al., 2013) is that subjects were asked to exert normal and tangential digit forces with different combinations of magnitude and direction during the “perceive and memorize” phase (see below).

#### Reference hand

As done in our previous study (Shibata et al., 2013), we measured three parameters of reference hand: (1) *length*, defined as the distance from the wrist crease to the tip of middle finger (mean ± SD: Group 1: 174.9 ± 9.7 mm; Group 2: 181.4 ± 8.1 mm); (2) *width*, defined as the distance between the radial prominence of the second metacarpo-phalangeal (*mcp*) joint and the ulnar prominence of the fifth *mcp* joint (mean ± SD: 80.5 ± 5.3 mm; Group 2: 84.2 ± 6.0 mm); and (3) *thumb-index distance*, defined as the distance between outstretched thumb and index fingertips (mean ± SD: 154 ± 12.6 mm; Group 2: 160.7 ± 14.3 mm). No outliers were found for any of these three parameters across subjects.

**Figure 3:**
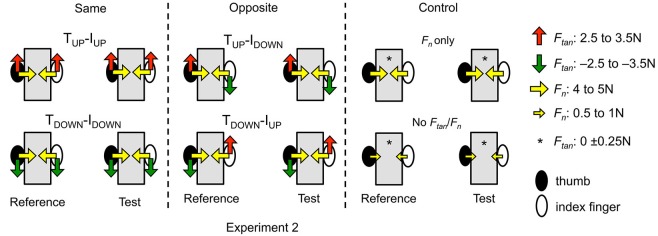
**Experimental conditions (Experiment 2).** The experimental conditions of Experiment 2 are shown in the same format as those shown in Figure 2 for Experiment 1. The only difference between Experiments 1 and 2 is that for the latter experiment, subjects were required to exert the same thumb and index fingertip normal and tangential forces across reference and test hands (see text for more details).

Subjects’ thumb and index fingertips of the reference hand were passively moved by an experimenter (*“passive d_y_ adjustment”* phase, Figure 2A) such that the CoPs of both digits on the graspable surface were at the same vertical height relative to the base of the object. Throughout the manuscript, we will refer to this fingertip position as “collinear” (*d_y_* = 0 mm; Figure 1B). During this procedure and while matching *d_y_* with the test hand (see below for details), subjects were instructed to extend the middle, ring, and little fingers to prevent them from contacting the handle (Figure 1B). The CoP and forces for each digit and the resultant *d_y_* of the reference hand was displayed on a second computer monitor that was not visible to the subject. Once an experimenter visually confirmed compliance of the desired hand posture and *d_y_*, a verbal cue was given to generate forces in one of six combinations of direction and magnitude (Figure 2B). Specifically, the reference hand exerted tangential force of thumb and index finger in either the same or opposite directions. When tangential forces were exerted in the same direction, both thumb and index finger exerted the tangential force upward (T_UP_-I_UP_) or downward (T_DOWN_-I_DOWN_) (“Same”; Figures 2B and 3, left column). When tangential forces were exerted in opposite directions, the thumb and index finger exerted the tangential force either upward and downward (T_UP_-I_DOWN_) or downward and upward, respectively (T_DOWN_-I_UP_) (“Opposite”; Figures 2B and 3, middle column). The range of the normal and tangential forces exerted by each digit of the reference hand was the same across these four experimental conditions (4–5 N and 2.5–3.5 N, respectively).

As these conditions always involve normal force of 4–5 N, subjects’ ability to match *d_y_* may potentially be affected by the combined effect of exerting normal and tangential forces. To isolate the effect of tangential force, we asked subjects to exert different magnitudes of normal force in two additional conditions that served as controls for the above-mentioned four conditions (“Control”; Figures 2B and 3, right column). In these control conditions, the tangential force was negligible (0 ± 0.25 N) and the normal force of the reference hand was either within the same range as for the above-mentioned conditions (4–5 N; “*F_n_* only” condition, Figure 2B, right column), or negligible (0.5–1 N; “No *F_n_*/*F_tan_*” condition, Figure 2B, right column). The lower bound of the normal force was required for accurate computation of digit CoP using the force sensor (Fu et al., 2010). To facilitate control of digit forces, subjects received visual feedback of digit normal and tangential forces on a computer monitor placed on the tabletop throughout each trial. Note that subjects were not given visual feedback of digit CoP throughout the experiment.

Upon confirmation of both of the above-described force and collinear CoP criteria, an auditory cue was given to subjects to start perceiving and memorizing the *d_y_* of the reference hand (“*perceive and memorize*”, Figure 2A). During this phase, subjects were required to maintain a given combination of digit normal and tangential forces as well as initial *d_y_* for 5 s within a tolerance window of ±3 mm from the collinear *d_y_*. If the digit CoPs shifted over the contact surface during the “perceive and memorize” phase and moved from their initial collinear placement (*d_y_* ≠ 0 mm), subjects were asked to relax the digits while an experimenter adjusted the digit CoPs to their original placement and the trial was re-started. If this adjustment had to be performed more than three times within a given trial, subjects were asked to completely relax the digits, release them from the sensor, and place the hand flat on the table with all digits straight, adducted, and with the palm in a pronated position before the trial could be re-started. Throughout the experiment, subjects were able to maintain the collinear *d_y_* within the ±3 mm tolerance window in 98.3% of all trials and the target force with the reference hand within the prescribed range in 98.0% of all trials. This “*perceive and memorize*” phase was terminated by an auditory cue so that subjects released the digits of reference hand from the handle and placed the hand flat on the table (“*relax*” phase, 10 s; Figure 2A). After this 10-s delay, another auditory cue was given to subjects to match the remembered reference hand *d_y_* using the same hand (“test hand”) within 10 s (see below for details). Note that the experimental setup, conditions, and procedures using the reference hand were identical for Experiments 1 and 2.

#### Test hand

Subjects were asked to actively place the thumb and index finger of the test hand to match the remembered *d_y_* within 10 s after making contact with the same handle (“*match*”, Figure 2A). During the “*match*” phase, subjects gave a verbal cue to the experimenter only when they could maintain digit forces within the target force range while matching the remembered *d_y_* using the test hand. Note that digit forces exerted by the test hand differed across Experiments 1 and 2. Specifically, during the “*match*” phase of Experiment 1 subjects were asked to reproduce *d_y_* while exerting negligible forces (0.5–1 N and 0 ± 0.25 N, respectively; Figure 2B). In contrast, for Experiment 2 subjects were asked to reproduce *d_y_* while also matching the forces they had exerted with the “reference” hand during “*perceive and memorize*” phase (Figure 3). Therefore, in Experiment 2, digit forces of the test hand were required to be the same as those exerted by the reference hand. Subjects controlled the digit forces using an online force gauge and values were shown separately for the tangential and normal forces of the thumb and index finger on a computer monitor. Throughout the experiment, subjects were able to maintain the target force with the test hand in 97.6% of all trials. The comparison between the Experiments 1 and 2 allowed us to study whether subjects’ ability to match the reference hand *d_y_* would be sensitive to whether digit forces, contact area, and skin deformation of the finger pad differ (Experiment 1) or are identical (Experiment 2) across reference and test hands. Note that both Experiments 1 and 2 included the Same, Opposite, and Control conditions (Figure 3).

After the subject’s verbal cue and when the force criteria were met, the experimenter gave a verbal cue to hold the *d_y_* and digit forces for 5 s during which CoPs of the test hand thumb and index finger were recorded (“*hold*”, Figure 2A). The trial was repeated if subjects did not give the verbal cue signaling attainment of the remembered *d_y_* or did not maintain digit forces within the target range during the “*match*” or “*hold*” phases. Finally, subjects were asked to release the test hand from the handle after another auditory cue was given.

Subjects practiced to control the required forces in all conditions for 10–20 min without being asked to match digit CoPs across reference and test hands. After the practice trials, at least 2 practice trials per condition (i.e., total of 12 practice trials) were given to subjects to familiarize themselves with the matching task. Note that subjects were not provided with feedback about matching performance during the practice or experimental trials. Subjects performed a total of 30 trials (5 trials × 6 experimental conditions). The order of presentation of experimental conditions was randomized across trials and subjects. Subjects were given rests every 10 trials or as appropriate to ensure that no fatigue occurred.

### Data processing

Force and torque data were acquired, recorded, and stored in a computer with a 12-bit A/D converter board (PCI-6225, National Instruments, Austin, TX; sampling frequency: 1 kHz) through a custom data acquisition interface (LabVIEW version 8.0, National Instruments). During data collection, force data were filtered online using a moving average filter every 50 samples over the 5-s duration of data recording for both reference and test hands. The filtered force data were then used for computing and displaying online normal and tangential force magnitudes and digit CoPs and *d_y_* using LabVIEW.

After data collection, CoP data for each digit were averaged within each trial and used to compute *d_y_* off-line with custom-written software (Matlab, The MathWorks, Natick, MA) for statistical analysis. The *d_y_* was defined as the vertical coordinate of thumb CoP minus the vertical coordinate of index finger CoP. Thus, positive and negative *d_y_* indicates that the thumb CoP is higher or lower relative to the index finger CoP, respectively. *Matching error* was defined as test hand *d_y_* during the “*hold*” phase minus reference hand *d_y_* during the “*perceive and memorize*” phase (Figure 2A). Note that in the present study, the reference hand *d_y_* was always 0 ± 3 mm. Matching error can be positive or negative, and thus takes into consideration whether subjects made an error not only in reproducing the distance between fingertip CoPs but also in their relative position. Specifically, positive and negative matching errors indicate that the test hand *d_y_* is positive and negative (i.e., the thumb CoP is higher and lower relative to the index finger CoP, respectively) compared to the reference hand *d_y_*.

### Statistical analysis

After computing matching errors and before performing statistical analyses, we determined whether there were outliers (data above or below three standard deviations of the mean) within each experimental condition per subject. As no outliers were found, all matching errors were included in statistical analyses.

We performed linear regression analysis on reference hand *d_y_* vs. test hand *d_y_* on trials (*n* = 5) from each experimental condition per subject. This analysis was performed to determine whether trial-to-trial deviations from the desired reference hand *d_y_* within ± 3 mm tolerance window induced systematic changes in test hand *d_y_*. Furthermore, to determine whether subjects’ matching performance varied systematically throughout the duration of the experiment, we also performed linear regression analysis on the matching error over 30 consecutive trials within subjects.

A mixed-design analysis of variance (ANOVA) was performed on matching errors in the control conditions (right column, Figures 2B and 3) with within-subject factor *Digit normal force* (2 levels: large, negligible) and *Experiment* as between-group factor (2 levels: Experiment 1, Experiment 2). The within-subject factor was used to analyze the effect of digit normal force magnitude on *d_y_* matching accuracy. The between-subject factor was used to test the effect of congruence of digit normal force between the reference and test hands on *d_y_* matching accuracy.

Matching errors in the Same and Opposite conditions (Figures 2A and 3, left and middle column) that were normalized to the errors in the “*F_n_* only” condition were analyzed using a mixed-design ANOVA with within-subject factors *Congruence of digit forces* (2 levels: Same, Opposite) and *Direction of tangential force* (2 levels: Up, Down), and *Experiment* as between-groups factor (Experiment 1, Experiment 2). The first within-subject factor was used to analyze the effect of all combinations of digit force direction on *d_y_* matching accuracy (Same: T_UP_-I_UP_ and T_DOWN_-I_DOWN_ vs. Opposite: T_UP_-I_DOWN_ and T_DOWN_-I_UP_). The second within-subject factor was used to examine the effect of tangential force direction on *d_y_* matching error. For this analysis, we used thumb tangential force direction to pool data in the “Up” and “Down” category (Up: T_UP_-I_UP_ and T_UP_-I_DOWN_ vs. Down: T_DOWN_-I_DOWN_ and T_DOWN_-I_UP_). For example, subjects might have made matching errors when thumb force was directed upward, but not downward. The between-subject factor (*Experiment*) was used to test the effect of having equivalent vs. different digit forces exerted by the reference and test hands on *d_y_* matching accuracy. This mixed-design ANOVA was performed to test the hypotheses that *(a)* the ability to match *d_y_*would be biased toward the direction of tangential force but only when the direction of tangential forces exerted by thumb and index finger was opposite; and *(b)* *d_y_* matching error would be greater in the Experiment 1 than Experiment 2 because the digit forces and skin deformation of the test hand differed from those of the reference hand. A *post-hoc* test was used to test the hypothesis that matching errors would be greater when the directions of tangential forces of the thumb and index finger were opposite than when they were the same. *Post-hoc* tests were run using paired sample *t*-tests with Bonferroni corrections when appropriate. Additionally, matching error for each experimental condition was analyzed by two-tailed one-sample *t*-tests to determine whether the mean matching error was significantly different from zero.

Sphericity assumptions were tested for all analyses of matching error (Mauchly’s sphericity test). When the sphericity assumptions were violated, we used Greenhouse-Geisser analysis (*p* < 0.01). Box’s test was used to test homogeneity of covariance (*p* > 0.05). All tests were performed at the *p* ≤ 0.05 significance level. Values in the text are reported as means ± standard error of the mean.

## Results

### Validation of experimental protocol

#### Effect of small trial-to-trial fluctuations in reference and test hand *d_y_*

Linear regression analysis on reference hand *d_y_* vs. test hand *d_y_* revealed that 93% of linear fits were not statistically significant (*p* > 0.05). The remaining 7% of linear fits that were statistically significant (*p* < 0.05) were characterized by inconsistent signs of regression coefficients. Therefore, the small trial-to-trial fluctuations in reference hand *d_y_* did not elicit systematic changes in test hand *d_y_*.

#### Effect of experimental duration on matching error

The linear regression analysis on the matching error over 30 trials within subjects revealed that all linear fits were not statistically significant (*p* > 0.05). This indicates that matching error did not systematically vary throughout the experiment and was independent of potential effects of experiment duration that might have induced fatigue, decrease in attention, or increasing familiarization with the task.

### Matching error

A mixed-design ANOVA on the matching errors in the two control conditions (Figures 2B and 3, right column) revealed no significant difference between matching performance in Experiments 1 and 2 (no main effect of *Experiment*: *F*_[1,28]_ = 0.467; *p* > 0.05) and between the “*F_n_* only” and “No *F_n_*/*F_tan_*” (no main effect of *Digit normal force*: *F*_[1,28]_ = 0.004; *p* > 0.05), and no significant interactions (*Digit normal force* × *Experiment*: *F*_[1,28]_ = 2.516; *p* > 0.05; Figure 5A). These results indicate that subjects’ ability to reproduce the reference *d_y_* with the test hand was not sensitive to whether reference and test hands exerted the same or different digit normal force. As matching error did not differ as a function of digit normal force in either experiment, the mean matching error from the “*F_n_* only” condition was used as a within-subject reference to normalize errors in the other experimental conditions characterized by the same normal force (4–5 N). The normalized matching error was defined as the mean matching error averaged within subjects in the Same and Opposite conditions minus the mean matching error from the “*F_n_* only” condition. This resulted in a “normalized matching error” denoting the effect of tangential force production only on *d_y_* matching error.

**Figure 4:**
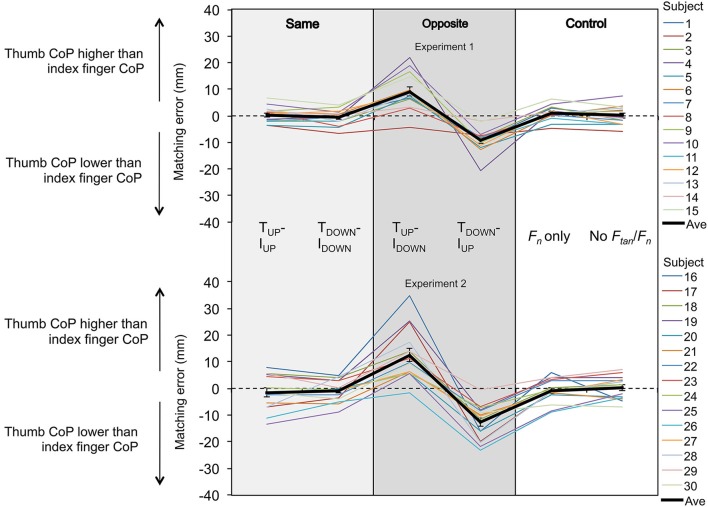
**Fingertip vertical distance: matching performance by individual subjects.** Mean matching errors averaged across five trials from each subject are shown as a function of experimental condition from Experiments 1 and 2 (top and bottom plots, respectively). Each subject data is color coded whereas the thick black line denotes the mean matching error averaged across 15 subjects ± standard error of the mean. For both experiments, positive and negative matching errors indicate that subjects reproduced remembered reference hand *d_y_* by placing the thumb CoP higher and lower, respectively, than the index finger CoP.

**Figure 5:**
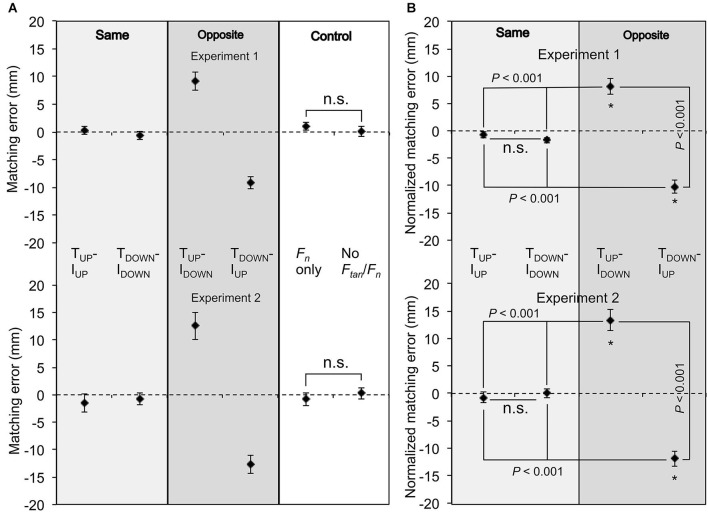
**Fingertip vertical distance: matching errors.** Matching errors were compared across experimental conditions and between experiments. **(A)** shows average matching error for Experiments 1 and 2 (top and bottom plots, respectively) across matching conditions. The mean matching error in the “*F_n_* only” condition was used as a reference to normalize the matching error in the Same and Opposite conditions (left and middle column, respectively; see text for more details). **(B)** shows average normalized matching error for the Experiments 1 and 2 (top and bottom plots, respectively) across matching conditions. For all panels, matching and normalized errors were averaged across all subjects (vertical bars denote SE). Asterisks denote significant differences (*p* < 0.05) from zero, whereas “n.s.” denote non-significant differences.

Figure 4 shows the matching error from each subject and the mean matching error averaged across all subjects for Experiments 1 and 2 (top and bottom plots, respectively). Matching errors made by each subject are connected by color-coded lines whereas the mean matching error averaged across all subjects is denoted by the thick black line. Matching errors were very small and similar across conditions where both digits exerted tangential forces in the same direction, indicating that subjects could reproduce fairly accurately a collinear digit fingertip position. The same result was found for experimental conditions where subject exerted only normal force or no tangential and normal forces (Control, Figure 4). However, matching error increased when digit tangential forces were exerted in opposite directions (T_UP_-I_DOWN_, T_DOWN_-I_UP_). For these experimental conditions, the direction of the error depended on whether a given digit exerted tangential force in the upward or downward direction. Specifically, for the T_UP_-I_DOWN_ condition, subject placed the thumb higher than the index fingertip, whereas for the T_DOWN_-I_UP_ subjects placed the index fingertip lower than the thumb. Overall, this trend of matching errors was similar across subjects and between experiments (top and bottom rows, Figure 4).

The results of the mixed-design ANOVA revealed a statistically significant difference in the normalized matching errors when comparing the conditions where thumb force was directed upward (T_UP_-I_UP_ and T_UP_-I_DOWN_) and downward (T_DOWN_-I_DOWN_ and T_DOWN_-I_UP_) (main effect: *Direction of tangential force*: *F*_[1,28]_ = 143.428; *p* < 0.001), but no significant difference when force direction of the thumb and index finger was the same or opposite (no main effect of *Congruence of digit forces*: *F*_[1,28]_ = 1.47; *p* > 0.05; Figure 5B). More importantly, we found a significant interaction between *Congruence of digit forces* and *Direction of tangential force* (*F*_[1,28]_ = 99.349; *p* < 0.001; Figure 5B). *Post hoc* paired *t-*tests with Bonferroni corrections found that subjects made significantly greater normalized matching errors when the force direction of the thumb and index finger was upward and downward (Opposite condition: T_UP_-I_DOWN_), respectively, than when it was the same (Same conditions: T_UP_-I_UP_ and T_DOWN_-I_DOWN_; *t*_[29]_ = −8.290, and −9.335, respectively; *p* < 0.001 for all conditions; adjusted *α* = 0.008; Figure 5B). Furthermore, subjects made greater absolute normalized matching errors when the force direction of the thumb and index finger was downward and upward (T_DOWN_-I_UP_) than when it was the same (T_UP_-I_UP_ and T_DOWN_-I_DOWN_; *t*_[29]_ = −12.320, and −9.288, respectively; *p* < 0.001 for all conditions; adjusted *α* = 0.008; Figure 5B). Furthermore, the normalized matching error in the T_UP_-I_DOWN_ condition was significantly different from that in the T_DOWN_-I_UP_ condition (*t*_[29]_ = −10.978; *p* < 0.001; adjusted *α* = 0.008; Figure 5B). No significant difference was found for pairwise comparison between the T_UP_-I_UP_ and T_DOWN_-I_DOWN_ conditions. These findings indicate that subjects’ ability to match remembered reference hand *d_y_* was sensitive to the congruence in the direction of tangential forces exerted by the thumb and index finger.

We also found a directional bias in *d_y_* matching errors. Specifically, subjects tended to make positive and negative matching errors in the T_UP_-I_DOWN_ and T_DOWN_-I_UP_ conditions, respectively (Figures 4 and 5B). The positive matching error denotes that subjects positioned the thumb CoP higher than index finger CoP when the tangential forces of thumb and index finger were directed upward and downward, respectively (T_UP_-I_DOWN_; Figures 4 and 5B), and vice versa for the T_DOWN_-I_UP_ condition. Two-tailed one-sample *t*-tests revealed that normalized matching errors were significantly different from zero when the direction of tangential digit forces in the reference hand was opposite, but not when it was the same (*p* < 0.001; Figure 5B). This indicates that subjects’ ability to match *d_y_* was biased toward the direction of tangential force, as indicated by the congruence in the vertical placement of each fingertip and the direction of the tangential force exerted by the same fingertip, but only when the direction of tangential forces was opposite.

Lastly, a mixed-design ANOVA confirmed that there was no statistically significant difference in the normalized matching errors between Experiments 1 and 2 (no main effect of *Experiment*: *F*_[1,28]_ = 3.77; *p* > 0.05) and no significant interactions with *Experiment* (*Congruence of digit forces* × *Experiment*: *F*_[1,28]_ = 0.803; *Direction of tangential force* × *Experiment*: *F*_[1,28]_ = 1.932; *Congruence of digit forces* ×*Direction of tangential force* × *Experiment*: *F*_[1,28]_ = 3.97; all *p* > 0.05). This indicates that subjects’ ability to match the reference hand *d_y_* was not dependent on equivalence in digit forces between reference and test hand.

## Discussion

We quantified the effects of motor commands responsible for generating digit forces on accuracy of sensorimotor transformation of the relative vertical distance between digit contact points. The main findings of this study are that accuracy in the sensorimotor transformation of vertical fingertip distance (1) is sensitive to whether tangential, but not normal, forces of thumb and index finger are produced in the same or opposite direction; and (2) is not sensitive to whether the hand used for matching fingertip distance exerts the same or different forces relative to those experienced during sensing. These results are discussed in the context of neural mechanisms underlying the sensorimotor transformation of digit position required for dexterous manipulation.

### Methodological considerations

The extent to which digit normal forces might affect matching horizontal fingertip distance between the contacts was not the focus of the present study and therefore was not investigated. Nevertheless, our findings indicate that generating digit normal forces *per se* does not affect the reproduction of relative vertical contact points. Similarly, with regard to potential effects of tangential digit forces exerted in the same direction (Same condition: T_UP_-I_UP_, T_DOWN_-I_DOWN_), we did not require subjects to match the height at which both fingertips had to be positioned relative to the object. Thus, subjects might have placed both digits higher or lower relative to the object when the direction of digit tangential forces was the same. However, the rationale for these experimental conditions was to rule out the possibility that voluntary motor commands for tangential force production—even when exerted in the same direction—could affect subjects’ ability to reproduce the relative vertical distance between contact points. As subjects could reproduce these points very accurately in the Same condition (Figure 5B), we conclude that the reproduction of the relative vertical distance between contact points was interfered with only when tangential digit forces were exerted in opposite direction, rather than by tangential or normal force production *per se*.

### Effects of motor commands on sensorimotor transformations

Biased matching errors found in the Opposite condition but not in the other conditions (see above) are accountable by the incongruent direction of digit tangential forces. Specifically, neither the exertion of digit normal forces alone (i.e., “*F_n_* only”) nor the congruent direction of digit tangential forces (i.e., Same condition: T_UP_-I_UP_, T_DOWN_-I_DOWN_) affected the reproduction of the remembered relative vertical distance between digit contact points. Consistent with our previous study (Shibata et al., 2013), we found that subjects can accurately reproduce relative vertical distance between contact points when the magnitude of neither tangential nor normal digit forces is significant (“No *F_tan_*/*F_n_*”, Figure 5A) and when significant normal digit forces only were exerted (“*F_n_* only”, Figure 5A).

This result supports our first hypothesis and confirmed such effects of voluntary motor commands on the sensorimotor transformations involved in matching fingertip distance as indicated by larger errors in the reproduced relative digit contact points when the tangential digit forces were exerted in opposite vs. same directions (Figure 5B). Importantly, the directionality of the matching errors in the present study was biased toward the direction of the voluntary motor commands, which is consistent with findings from previous studies (Gandevia et al., 2006; Smith et al., 2009; see below). Specifically, we found that subjects erroneously placed the thumb higher than the index finger (i.e., positive matching error) when the upward and downward tangential forces were exerted by the thumb and index finger, respective (T_UP_-I_DOWN_), and vice versa for the T_DOWN_-I_UP_ (Figures 4 and 5). We also found that, contrary to our second hypothesis, the magnitude of the matching error was the same regardless of whether subjects were asked to exert negligible force or match digit force exerted with the Reference hand using the Test hand (Experiments 1 and 2, respectively). This result indicates that the mismatch in digit forces exerted by Reference and Test hands was not the primary cause of bias in matching error, and further suggests that this might have been primarily driven by a conflict between motor commands and sensory feedback during the *“perceive and memorize”* phase (see below).

Note that matching tasks in previous studies (Gandevia, 1987; Gandevia et al., 2006; Smith et al., 2009) required subjects to indicate a joint angle using the contralateral limb relative to the one used as a “reference”, whereas our task required subjects to match the relative vertical digit contact points using the same hand. Thus, our task might be considered more complex due to the requirement of integrating the perceived spatial relation between two contact points to estimate their vertical distance. Moreover, subjects in the present study were required to perceive and memorize the contact points, retain the perceived fingertip distance for a short period of time, and then retrieve and use the memorized fingertip distance to place the digits at the remembered relative locations. In contrast, the above-cited previous work did not require subjects to memorize a given joint angle. Despite differences in matching task between previous work and the present study, we found a similar phenomenon: voluntary motor commands associated with force production affect the directionality of the matching error when the directions of digit forces were opposite (Opposite condition: T_UP_-I_DOWN_, T_DOWN_-I_UP_).

Centrally-generated voluntary motor commands for force production are thought to affect processing of somatosensory afferent signals to estimate limb joint angle (Gandevia, 1987; Gandevia et al., 2006; Smith et al., 2009; for review see Proske and Gandevia, 2012). This proposition is consistent with the framework of internal forward models in which a copy of motor commands is used to predict sensory consequences of motor commands, which are then compared with incoming sensory feedback to estimate sensory state in the immediate future (Wolpert et al., 1995; Kawato, 1999). In the present study, voluntary motor commands responsible for digit tangential force production in opposite directions and in absence of friction would have resulted in increasing the relative vertical distance between the fingertips. It should also be noted that during digit force exertion, afferent discharge from skin, muscle, and tendon receptors should have accurately encoded the relative position of the finger pads. Therefore, the fact that matching error was highly sensitive to the pattern of digit tangential force direction implies that the prediction of sensory consequences of force generation overrode sensory feedback from the finger pads. Thus, fingertip distance reproduction was distorted in a way that resembled the relative fingertip position resulting from motor commands—had the fingertip being allowed to move—rather than the actual distance as encoded by somatosensory receptors.

### When did sensorimotor transformation errors occur?

Throughout our matching task, errors in sensorimotor transformations might have been induced by four non-mutually exclusive factors: (1) inaccurate perception of the relative vertical contact points; (2) time-dependent decay of memory of perceived fingertip distance; (3) inaccurate memory retrieval; and/or (4) inaccurate motor commands for placing the digits to the remembered contact points. We propose that the last three factors did not play a significant role in causing the matching error. This interpretation is based on the similarity in the bias effect on matching error found by the above-cited psychophysical studies (Gandevia et al., 2006; Smith et al., 2009) despite major task differences. Specifically, this previous work did not incorporate a memory component or motor commands for reproducing joint angle. In contrast, our task required subjects to store, retain, and retrieve memory of relative contact points, and send motor commands using the same hand for reproducing the remembered relative contact points. Therefore, we conclude that the sensorimotor transformation errors likely occurred when subjects perceived and memorized the relative vertical contact points.

### Dexterous manipulation: motor commands for positioning digits and generating forces

Behavioral evidence indicates that subjects can accurately modulate digit forces as a function of variable digit placement while exerting a torque, thus indicating successful sensorimotor transformations (Fu et al., 2010). Importantly, such modulation is found following exertion of normal and tangential force up to the instant of object lift-off, as well as when digit tangential forces are exerted in opposite direction to generate a torque. In contrast, the present study shows that sensorimotor transformations are inaccurate when digit tangential forces are exerted in opposite directions. However, several factors might enable successful sensorimotor transformations in dexterous manipulation tasks while preventing them in our psychophysical task. First, visual feedback of contact points prior and following contact might wash out the bias induced by voluntary commands of digit forces, whereas visual feedback of the hand was prevented in our study. Second, manipulation tasks involve active digit placement on objects, whereas in our experiment subjects’ fingertips were passively placed on the object.

Many studies have shown that an estimation of limb endpoint relative to the body after active reaching movements is more precise than after passive reaching movements (Adamovich et al., 1998; Gritsenko et al., 2007; Fuentes and Bastian, 2010; Bhanpuri et al., 2013). For the active movement case, subjects voluntarily moved their arm to a target, whereas in the passive movement condition their arm was passively moved by a robot. In contrast, other studies have shown no difference in the estimation of limb endpoint between active and passive reaching movements (Jones et al., 2010; Capaday et al., 2013). Moreover, haptic sensitivity for discriminating between two curved paths of the arm movement was similar between the active and passive reaching movements (Sciutti et al., 2010). Furthermore, a difference in accuracy in the perception of the curved path (Sciutti et al., 2010) and joint angle during the reaching movement (Gritsenko et al., 2007) between the active and passive movements was most pronounced as the movement amplitude increased. These findings indicate that voluntary motor commands for force production and positioning the arm during the active movement may or may not facilitate the estimation of the limb endpoint. However, a recent study (Bhanpuri et al., 2013) has shown that estimation of hand endpoint after an active arm movement was more accurate when a physical contact of the hand to stop the arm movement could be predicted as a consequence of the movement. Hence, the estimation of the endpoint was likely facilitated by not only voluntary motor commands for the arm movement, but also by the expected sensory consequences, i.e., the predictable physical contact in the cited study. Further investigation, however, is needed to address potential effects of voluntary digit movement on sensing relative contact points for execution of dexterous manipulation.

## Conclusions

The present errors in somatosensory-motor transformations of relative vertical contact points indicate that voluntary commands responsible for generating digit forces in opposite direction affect the accuracy with which perceived fingertip distance can be reproduced. We speculate that the CNS implements voluntary motor commands for position and force production as well as predictable sensory consequences for successful sensorimotor transformations required for object manipulation. The extent to which predictable sensory consequences from motor commands for digit position and force underlie accurate force modulation during a dexterous manipulation is the subject of ongoing investigation.

## Conflict of interest statement

The authors declare that the research was conducted in the absence of any commercial or financial relationships that could be construed as a potential conflict of interest.
